# NBR1-p62-Nrf2 mediates the anti-pulmonary fibrosis effects of protodioscin

**DOI:** 10.1186/s13020-024-00930-0

**Published:** 2024-04-08

**Authors:** Qian Zeng, Bin-bin Wen, Xin Liu, Yong-yu Luo, Zhen-gang Hu, Lei Huang, Xiao-hua Zhang, Xiao-ting Huang, Ting-ting Zhou, Xiao-xue Sang, Yu-yang Luo, Da-yan Xiong, Zi-qiang Luo, Wei Liu, Si-yuan Tang

**Affiliations:** 1 Xiangya Nursing School, Central South University, 172 Tongzipo Road, Changsha, 410013 Hunan China; 2 The Orthopedics Hospital of Traditional Chinese Medicine Zhuzhou City, Zhuzhou, Hunan China; 3 Guiyang Second People’s Hospital, Guiyang, Guizhou China; 4 Xiangya Hospital, Central South University, Changsha, Hunan China; 5 Hunan Prevention and Treatment Institute for Occupational Diseases, Changsha, China; 6 Xiangya School of Medicine, Central South University, Changsha, Hunan China

**Keywords:** Protodioscin, Anti-pulmonary fibrosis, NBR1-p62-Nrf2 pathway, Oxidative stress

## Abstract

**Background:**

Idiopathic pulmonary fibrosis is a persistent disease of the lung interstitium for which there is no efficacious pharmacological therapy. Protodioscin, a steroidal saponin, possesses diverse pharmacological properties; however, its function in pulmonary fibrosis is yet to be established. Hence, in this investigation, it was attempted to figure out the anti-pulmonary fibrosis influences of protodioscin and its pharmacological properties related to oxidative stress.

**Methods:**

A mouse lung fibrosis model was generated using tracheal injections of bleomycin, followed by intraperitoneal injection of different concentrations of protodioscin, and the levels of oxidative stress and fibrosis were detected in the lungs. Multiple fibroblasts were treated with TGF-β to induce their transition to myofibroblasts. It was attempted to quantify myofibroblast markers’ expression levels and reactive oxygen species levels as well as Nrf2 activation after co-incubation of TGF-β with fibroblasts and different concentrations of protodioscin. The influence of protodioscin on the expression and phosphorylation of p62, which is associated with Nrf2 activation, were detected, and p62 related genes were predicted by STRING database. The effects of Nrf2 inhibitor or silencing of the Nrf2, p62 and NBR1 genes, respectively, on the activation of Nrf2 by protodioscin were examined. The associations between p62, NBR1, and Keap1 in the activation of Nrf2 by protodioscin was demonstrated using a co-IP assay. Nrf2 inhibitor were used when protodioscin was treated in mice with pulmonary fibrosis and lung tissue fibrosis and oxidative stress levels were detected.

**Results:**

In vivo, protodioscin decreased the levels of fibrosis markers and oxidative stress markers and activated Nrf2 in mice with pulmonary fibrosis, and these effects were inhibited by Nrf2 inhibitor. In vitro, protodioscin decreased the levels of myofibroblast markers and oxidative stress markers during myofibroblast transition and promoted Nrf2 downstream gene expression, with reversal of these effects after Nrf2, p62 and NBR1 genes were silenced or Nrf2 inhibitors were used, respectively. Protodioscin promoted the binding of NBR1 to p62 and Keap1, thereby reducing Keap1-Nrf2 binding.

**Conclusion:**

The NBR1-p62-Nrf2 axis is targeted by protodioscin to reduce oxidative stress and inhibit pulmonary fibrosis.

**Graphical Abstract:**

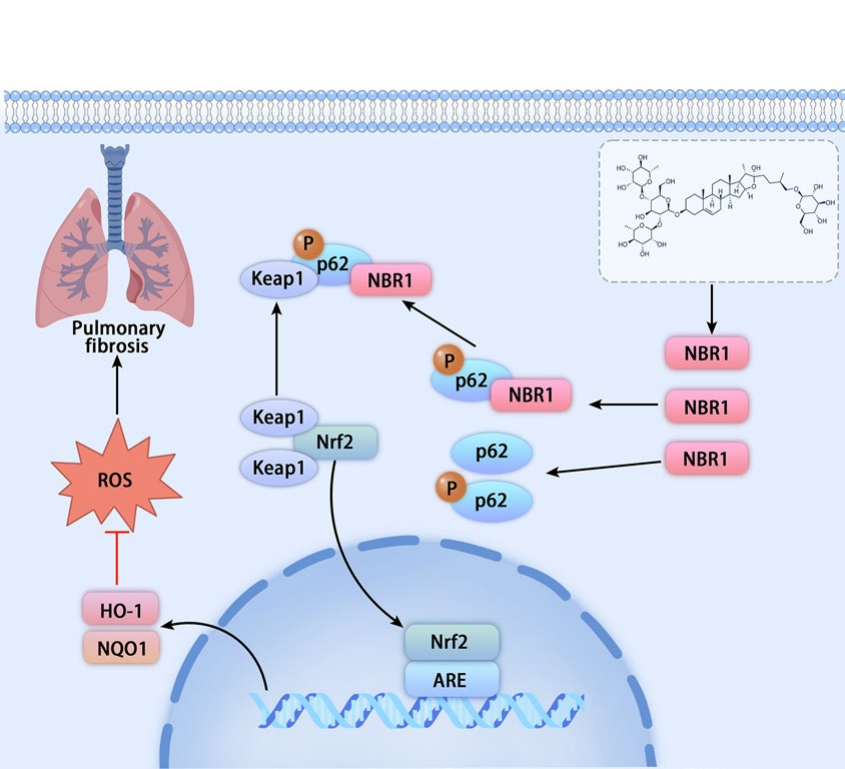

**Supplementary Information:**

The online version contains supplementary material available at 10.1186/s13020-024-00930-0.

## Background

As a persistent, progressive and fibrotic lung disease, idiopathic pulmonary fibrosis (IPF) typically manifests with disrupted alveolar structures, the replacement of healthy tissues with a modified extracellular matrix as well as an abnormal buildup of fibrous tissues in the lung parenchyma. As a result, lung compliance decreases and gas exchange is hindered, with disease progression subsequently leading to respiratory failure and eventual death [1]. Statistically, IPF’s incidence and prevalence have been increasing globally every year, and both rise with age [2]. Treatment of pulmonary fibrosis involves the use of two FDA-approved drugs, namely Pirfenidone and Nintedanib, which can delay progression of the disease and reduce the risk of death. However, in addition to their tolerability issues and adverse effects, these 2 drugs are also unable to halt or reverse IPF’s progression [3, 4]. Therefore, it is important to identify drugs targeting IPF.

The mechanisms underlying the development of IPF are currently unknown. Fibroblasts can transition to myofibroblasts in response to pro-fibrotic factors is an important pathological alteration in the course of IPF, and hence, most studies have tended to focus on therapeutic approaches targeting myofibroblasts [5, 6]. Although the pathogenesis of IPF has not yet been explored in detail, oxidative stress is known to be closely involved in its development [7]. Indeed, oxidative stress promotes myofibroblasts formation through enhanced secretion of different cytokines such as TGF-β. The increased numbers of myofibroblasts lead to extracellular matrix production, thereby fostering fibrosis [8]. Therefore, interventions against oxidative stress have the potential to inhibit myofibroblast formation, thus inhibiting the development of pulmonary fibrosis.

Various substances play key roles within the body’s antioxidant defense mechanism to counteract oxidative stress-induced damage. In particular, nuclear factor erythroid 2-related factor 2 (Nrf2) is considered a crucial transcription factor responsible for increasing the expression of antioxidant genes [9]. Thus, Nrf2 activation is pivotal in countering IPF. For instance, in vivo, activation of Nrf2 inhibits fibrotic progression in various animal models of pulmonary fibrosis [10–12]. In vitro, activation of Nrf2 leads to higher expression of antioxidant genes, decreased reactive oxygen species (ROS) levels as well as increased dedifferentiation of myofibroblasts in fibroblasts cultured from both control subjects and cases with idiopathic pulmonary fibrosis [13–15]. These findings present Nrf2 as a potential target for treating IPF and other oxidative stress-induced fibrotic diseases.

Nrf2 activation is regulated by various factors through classical and non-classical mechanisms [16]. The classical mechanism of Nrf2 activation involves oxidative stress and electrophilic compounds. This involves the oxidation of specific cysteine residues in Kelch-like ECH-associated protein 1 (Keap1), leading to decreased ubiquitylation of Nrf2, promoting its activation and translocation to the nucleus. The non-classical mechanism of Nrf2 activation involves several proteins, including p62/Sqstm1, p21, DPP3, and WTX. These proteins can directly interact with Keap1 to disrupt the Nrf2-Keap1 complex and decrease Nrf2 ubiquitination, resulting in increased nuclear translocation and activation. In fact, studies have shown that p62, in particular, is crucial for Nrf2 activation in various diseases [17–19]. It acts as a target and regulates positive feedback within the Nrf2-antioxidant response element transcriptional pathway. An interactive relationship exists between p62 and Nrf2. Initially, p62 binds only weakly to Keap1, but this interaction strengthens significantly after phosphorylation. This leads to the release of Nrf2, increasing its levels in the cytoplasm and enhancing transfer to the nucleus. Consequently, this effect amplifies the transcription of antioxidant genes, namely quinone 1 (NQO1), NAD(P)H dehydrogenase and heme oxygenase 1 (HO-1) which lie downstream of Nrf2 while increasing p62 expression. In essence, p62 regulates Nrf2 activation and p62 expression, forming a positive feedback loop [20, 21]. Nevertheless, the detailed mechanisms and factors affecting the activation of Nrf2 by p62 have not yet been fully explored.

Protodioscin is a steroidal saponin derived from the rhizome of the Chinese herb *Dioscorea collettii var. hypoglauca* [22]. This compound is also present in plants such as asparagus, yam, and fenugreek. Protodioscin exhibits a diverse array of pharmacological effects, including anticancer, anti-inflammatory, hypoglycaemic, and anti-injury effects [23–25]. However, its effects on fibrosis are currently unknown. In this study, we explored its anti-pulmonary fibrosis effects for the first time and investigated its pharmacological effects in detail from the perspective of anti-oxidative stress. Our study further advances the understanding of the pharmacological effects of protodioscin and provides a new reference for the pharmacological treatment of IPF.

## Materials and methods

### Animals and animal experimentation

The Laboratory Animal Welfare and Ethics Committee of Central South University attempted to authorize the study protocol, with IACUC number of CSU-2022-0001-0196, ensuring compliance with the ethical principles and welfare standards applicable to laboratory animals.

C57BL/6 mice and SD rats were acquired from the Department of Laboratory Zoology, Central South University and kept at the Central South University Department of Laboratory Zoology in a specific pathogen-free environment. Mice were acclimated to feeding for one week before lung fibrosis modelling and treatment. Mice were subjected to anesthesia utilizing sodium pentobarbital, and 50 µL of bleomycin (BLM; 3 mg/kg; Nippon Kayaku) was administered intratracheally, while controls received a corresponding volume of saline. Protodioscin (Solarbio) was diluted in a solvent consisting of saline, 5% Tween 80 (Macklin) (v/v), 40% polyethene glycol 300 (Macklin) and 5% dimethyl sulfoxide (Macklin).

To assess the therapeutic effect of protodioscin, 10 mice were randomly chosen from the total of 110 mice as the control group and they were injected with saline via the trachea before receiving intraperitoneal solvent injections on days 15–28. The remaining cohort of mice was subjected to the modeling procedure, employing a stratified sampling approach on day 14 based on individual body weights. Thereafter, an equivalent number of mice were systematically allocated to four distinct groups during the period spanning days 15 to 28. These groups comprised the low-dose treatment group, injected intraperitoneally with 1 mg/kg protodioscin; the medium-dose treatment group, injected intraperitoneally with 5 mg/kg protodioscin; the high-dose treatment group, injected intraperitoneally with 20 mg/kg protodioscin; the pulmonary fibrosis group, injected intraperitoneally with solvent. On day 29, all mice were anaesthetised with sodium pentobarbital, euthanised by femoral artery bloodletting, and lung tissues were removed for subsequent experiments.

To determine how a Nrf2 inhibitor (ML385) impacts the efficacy of protodioscin, 10 randomly-selected mice out of 100 were used as controls, and these were intratracheally injected with saline, followed by intraperitoneal solvent injections on days 15–28. The remaining animals were used as models, and for this purpose, they were stratified on day 14 according to their body weight before assigning an equal number of mice to the following three groups: in the specified experimental regimen, the high-dose cohort was administered intraperitoneal injections of 20 mg/kg protodioscin from days 15 to 28. Concurrently, the pulmonary fibrosis group underwent intraperitoneal injections of the designated solvent over the same duration. Moreover, the ML385 group received a distinctive treatment involving intraperitoneal injections of 30 mg/kg ML385 attained from Selleck Chemicals, followed by subsequent intraperitoneal injections of 20 mg/kg protodioscin three hours later. This intricate administration protocol was meticulously adhered to throughout the experimental timeline. On day 29, all mice were anaesthetised with sodium pentobarbital, euthanised by femoral artery bloodletting, and lung tissues were removed for subsequent experiments.

### Extraction and culture of primary lung fibroblasts

After euthanasia of mice or rats, their thoracic cavities were exposed, and their lung tissues were rinsed with ice-cold PBS supplemented with 1% penicillin–streptomycin solution (Procell). The tissues were then cut into cubes and incubated with 1 mg/mL type I collagenase (Gibco) for 1h at 37 ℃ in a water bath. After centrifugation, the lung tissue was resuspended prior to filtration through a 70-µm filter. The resulting filtrate was centrifuged and after resuspension in red blood cell lysate (Solarbio), a 5-min incubation was performed on ice. The above-described procedure was reiterated by passing the amalgam through a 40-µm filter, centrifugation, and subsequent reconstitution in high-glucose Dulbecco's Modified Eagle Medium (Procell). This medium was supplemented with 1% penicillin–streptomycin solution (Procell) and 20% fetal bovine serum (FBS; Biological Industries). Primary lung fibroblasts, spanning the 3–7 generation range, were utilized for all experimental protocols. Ham's F-12 K medium attained from ProCell, inclusive of 1% penicillin–streptomycin solution and 10% FBS, was utilized for the cultivation of human fetal lung fibroblast 1 cells (HFL-1) attained from ProCell. Incubated under optimal conditions at 37 ℃ in a humidified incubator, these cell cultures were maintained in an environment with 5% carbon dioxide.

### Hematoxylin and eosin (HE) and Masson staining, and Ashcroft scoring

The mouse lung tissues were subjected to thorough fixation utilizing 4% paraformaldehyde attained from Servicebio, and it was attempted to meticulously embed them in paraffin and section the tissues. These sections were thereafter subjected to staining procedures employing HE and Masson staining solutions, which both attained from Servicebio. The severity of lung fibres in each mouse was scored according to Ashcroft’s method [26].

### Immunohistochemistry

Xylene was used to de-paraffinize paraffin sections and after ethanol-based dehydration and subsequent antigen repair, the membranes were lysed with 0.5% Triton X-100 (Servicebio). Endogenous peroxidase was also blocked by covering the tissues with an endogenous peroxidase blocker (Beyotime Biotechnology). It was attempted to carry out 30-min incubation with goat serum particularly at room temperature, after which overnight incubation of tissues was undertaken at 4 ℃ with 8-Hydroxy-2-deoxyguanosine (8-OHdG; Servicebio), col-I (Proteintech) and α-SMA (Proteintech) antibodies. A 1-h incubation was then performed at room temperature with goat anti-rabbit IgG monoclonal antibody (Proteintech) prior to staining with a DAB chromogenic solution and subsequently, with haematoxylin.

### Measurement of hydroxyproline

The hydroxyproline assay kit attained from the Nanjing Institute of Construction Biotechnology, was employed in accordance with the provided guidelines. Mouse lung tissue underwent homogenization, followed by the sequential addition of various reagents. Subsequent centrifugation particularly at 3500 rpm for 10 min resulted in the supernatant, and it was attempted to transfer it to a 96-well plate for absorbance measurements at 550 nm. These recorded values were subsequently employed in the calculation of hydroxyproline content.

### Lung tissue ROS assay

A histochemical pen was used to draw circles around frozen sections before the introduction of a fluorescent quencher. After adding ROS staining solution into the circle, a 30-min incubation was performed at 37 ℃. After staining with DAPI for 10 min at room temperature, the sections were sealed with fluorescence quenching sealer.

### Malondialdehyde (MDA) and glutathione (GSH) levels and superoxide dismutase (SOD) activity measurements

Assay kits for glutathione, superoxide dismutase, and malondialdehyde were obtained from the Nanjing Institute of Construction Biotechnology and used following the provided directions. Mouse lung tissues were homogenised, various reagents were added sequentially, and the supernatants were collected after centrifugation to measure the absorbance at the corresponding wavelengths; SOD activity as well as GSH and MDA content were finally obtained by calculation.

### Determination of cell viability and cell supernatant lactate dehydrogenase (LDH)

Cell Counting Kit-8 (CKK-8) assays were performed using a kit (Elabscience) in accordance with the provided directions to detect cell viability, while determining lactate dehydrogenase level particularly in the cell supernatants was undertaken through a lactate dehydrogenase assay kit (Nanjing Institute of Construction Biotechnology). It was attempted to plate the cells onto 96-well plates and subjected to a 24-h incubation period with varying concentrations of protodioscin. Thereafter, the detection reagent was introduced, and the incubation of cells was subsequently undertaken at 37 ℃. The absorbance values at 450 nm for each well were then recorded.

### Western blotting (WB)

The extraction of total protein from mouse lung tissues or cells involved the utilization of RIPA lysis buffer containing protease and phosphatase inhibitors, including phenylmethylsulfonyl fluoride. Subsequent determination of the total protein concentration was undertaken through a BCA kit that was attained from Cwbio. Thereafter, the proteins were electrophoresed on 10% SDS-PAGE gels followed by transfer to PVDF membranes. The membranes were blocked with 5% skim milk, followed by incubation with antibodies against β-actin, α-SMA, Collagen I, Fibronectin 1 (FN1), CTGF, HO-1, NQO1, Nrf2, Lamin B1, p62, Phospho-P62 (Ser349), Keap1, NBR1, and PRKCI (all from Proteintech) overnight at 4 ℃. The 2-h incubation of membranes was then undertaken particularly at room temperature with goat anti-mouse or goat anti-rabbit IgG monoclonal antibodies, with the level of protein expression eventually visualized and assessed using an ECL developer.

### Total RNA extraction and quantitative reverse-transcriptase polymerase chain reaction (qRT-PCR)

Upon completion of total RNA extraction from both lung tissues and cells using TRIzol reagent attained from Thermo Fisher Scientific, reverse transcription into cDNA was undertaken through a reverse transcription kit attained from Thermo Fisher Scientific. Subsequently, it was attempted to carry out qRT-PCR on a Bio-Rad CFX96 Touch Real-time PCR Detection System, which attained from Bio-Rad, on the basis of the amplification conditions outlined below: 2-min initial denaturation at 95 ℃, followed by 40 cycles, each comprising a 3-s denaturation at 95 ℃ and a 30-s extension at 60 ℃. The reaction was completed with a melting curve of 60–95 ℃. Table 1 lists the different primers (Sangon Biotech) used in the experiment.

**Table 1:** Primers used in the experiment.

Gene	Forward [5'-3']	Reverse [5'-3']
Mouse collagen I	GAGCGGAGAGTACTGGATCG	GCTTCTTTTCCTTGGGGTTC
Mouse α-SMA	TGGCTATTCAGGCTGTGCTGTC	CAATCTCACGCTCGGCAGTAGT
Mouse β-Actin	GTGCTATGTTGCTCTAGACTTCG	ATGCCACAGGATTCCATACC
Mouse FN1	AGTGGCTGAAGTCGCAAGGAAAC	TAAGTCTGGGTCACGGCTGTCTC
Mouse CTGF	CACCGCACAGAACCACCACTC	AATGGCAGGCACAGGTCTTGATG
Rat β-Actin	TGTCACCAACTGGGACGATA	GGGGTGTTGAAGGTCTCAAA
Rat α-SMA	GCGTGGCTATTCCTTCGTGACTAC	CATCAGGCAGTTCGTAGCTCTTCTC
Rat collagen I	TGTTGGTCCTGCTGGCAAGAATG	GTCACCTTGTTCGCCTGTCTCAC
Rat FN1	AGGCACAAGGTCCGAGAAGAGG	CATGAGTCATCCGTAGGCTGGTTC
Rat CTGF	CACCGCACAGAACCACCACAC	AATGGCAGGCACAGGTCTTGATG
Human β-Actin	CCTGGCACCCAGCACAAT	GGGCCGGACTCGTCATAC
Human α-SMA	TCCGGAGCGAAATACTCTG	CCCGGCTTCATCGTATTCCT
Human collagen I	CCACCAATCACCTGCGTACA	CACGTCATCGCACAACACCT
Human FN1	GGCGACAGGACGGACATCTTTG	GGCACAAGGCACCATTGGAATTTC
Human CTGF	CGGGAAATGCTGCGAGGAGTG	GGTCTGGGCCAAACGTGTCTTC
Mouse Nrf2	AGTCCAGAAGCCAAACTGACAGAAG	GGAGAGGATGCTGCTGAAGGAATC
Mouse HO-1	ACCGCCTTCCTGCTCAACATTG	CTCTGACGAAGTGACGCCATCTG
Mouse NQO1	GCGAGAAGAGCCCTGATTGTACTG	AGCCTCTACAGCAGCCTCCTTC
Mouse p62	AGGAGGAGACGATGACTGGACAC	TTGGTCTGTAGGAGCCTGGTGAG
Mouse NBR1	AACAGGACTCGCAAACAGCAGAC	GTACGCAGAGGCAGCAACAGAC
Mouse PRKCI	TGACTACGGCATGTGTAAGGAAGG	CCAGTCAACGCTGAAGCCATAATC
Mouse WDFY3	TCACCAGCAGCCGATGTAAGC	GAAGAGACCACCGTTCCGCTAG
Mouse CYLD	TTCCAGGAGTTGTACGCTTCAGAG	AACCTTGACCACGACCTTCTTCC
Mouse TRAF6	ACTGCCCAACAGCTCCAATCC	CAAGTGTCGTGCCAAGTGATTCC

### Cellular reactive oxygen species assay

The 30-min incubation of cells was undertaken particularly at 37 ℃ with the ROS probe DCFH-DA attained from Beyotime Biotechnology. Subsequently, the cells underwent two additional washes with DMEM. The ensuing procedures involved either observation under a fluorescence microscope or collection for flow cytometry.

### SiRNA transfection

After the cell seeding plate, when the cell density reached 60–80%, control SiRNA, p62 SiRNA, Nrf2 siRNA (Santa Cruz) or NBR1 siRNA (Ribobio) was mixed with Lipofectamine 2000 as the transfection reagent (Invitrogen) and incubated for 12 h to detect the transfection effect or for subsequent intervention.

### Co-immunoprecipitation assays

An agarose Co-immunoprecipitation Assays (co-IP) kit (ABBKINE) was used as instructed by the manufacturer. Collected cells were lysed before determining the protein concentration. Immunoprecipitation was performed after removing non-specific binding and detected by WB.

### Statistical analysis

It was attempted to carry out statistical analysis through GraphPad Prism 8.3.1 software. The expression of data was undertaken in form of mean ± standard deviation, and pairwise comparisons were carried out using t-test. For the purpose of making comparisons involving multiple groups, one-way ANOVA, followed by Tukey's test was employed. A *P* threshold below 0.05 was suggestive of statistical significance.

## Results

### Protodioscin mitigates bleomycin-induced pulmonary fibrosis in mice

**Fig. 1:**
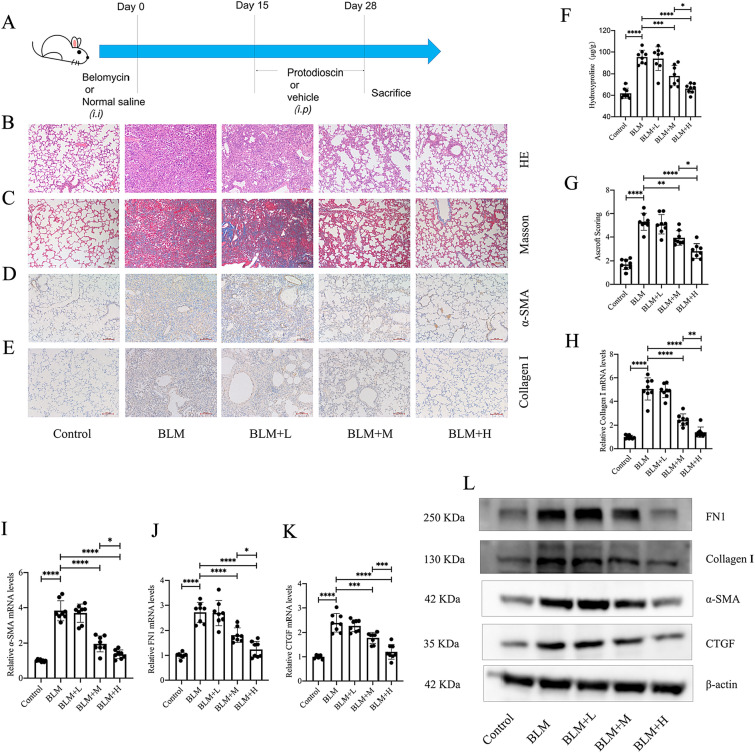
Protodioscin ameliorated pathological structural changes in lung tissues and decreased the levels of lung fibrosis markers in mouse pulmonary fibrosis. Tracheal injection of bleomycin was used to construct a mouse lung fibrosis model, and intraperitoneal injections of low (1 mg/kg), medium (5 mg/kg), and high (20 mg/kg) doses of protodioscin were administered to observe its effects on lung fibrosis (**A**). Alterations in lung tissue structures and the extracellular matrix were observed after HE and Masson staining (magnification × 100) (**B**, **C**). The extent of lung fibrosis in the different groups was determined by the Ashcroft score (**G**). Lung tissue contents of hydroxyproline were assessed by biochemical methods (**F**). The positive expression areas of col-I and α-SMA were analyzed by immunohistochemical methods tissues (magnification × 100) (**D**, **E**). The mRNA expression levels of *α-SMA*, *Col1a1*, *FN1* and *CTGF* expression in lung tissues were quantified by qRT-PCR (**H**–**K**). WB was undertaken to analyze the levels of col-I and α-SMA proteins in lung tissue (**L**). BLM + H indicates high-dose treatment group; BLM + M indicates medium-dose treatment group; BLM + L indicates low-dose treatment group; BLM indicates lung fibrosis model group; Control indicates control group. Expression of data was undertaken in form of mean ± standard deviation. The sample size, particularly per group, was eight, * *P* < 0.05; *It is suggestive of statistical significance at the *P* threshold below 0.05; **Denotes heightened significance at the *P* threshold below 0.01; ***Reflects substantial significance at the *P* threshold below 0.001; ****Demonstrates remarkable significance at the *P* threshold below 0.0001

The therapeutic effects of protodioscin on mouse pulmonary fibrosis were observed through intraperitoneal injections of high, medium, and low doses of protodioscin from day 15 to 28 after administering bleomycin via tracheal injection. This period represents the stage of gradual exacerbation of pulmonary fibrosis in mice (Fig. 1A). HE and Masson staining revealed significantly improved alveolar structures as well as decreased extracellular matrix accumulation in fibrotic mice when protodioscin was administered at high and medium doses (Fig. 1B, C), and this was in accordance with the results of Ashcroft's score (Fig. 1G). In addition, at these two doses, there was also a significant reduction in the hydroxyproline content of the lung tissues (Fig. 1F). Immunohistochemical data further indicated that protodioscin reduced the positivities fibrotic lung tissue (Fig. 1D, E). The mRNA and protein expression levels of col-I, FN1, CTGF and α-SMA in the lung tissues were also significantly reduced at medium and high doses of protodioscin (Fig. 1H–L). The effect of high protodioscin doses on each index was more evident than that of medium doses. These findings suggest that protodioscin can exert significant therapeutic effects on bleomycin-induced pulmonary fibrosis in mice.

### Protodioscin reduces oxidative stress in fibrotic mouse lung tissue and activates Nrf2

**Fig. 2:**
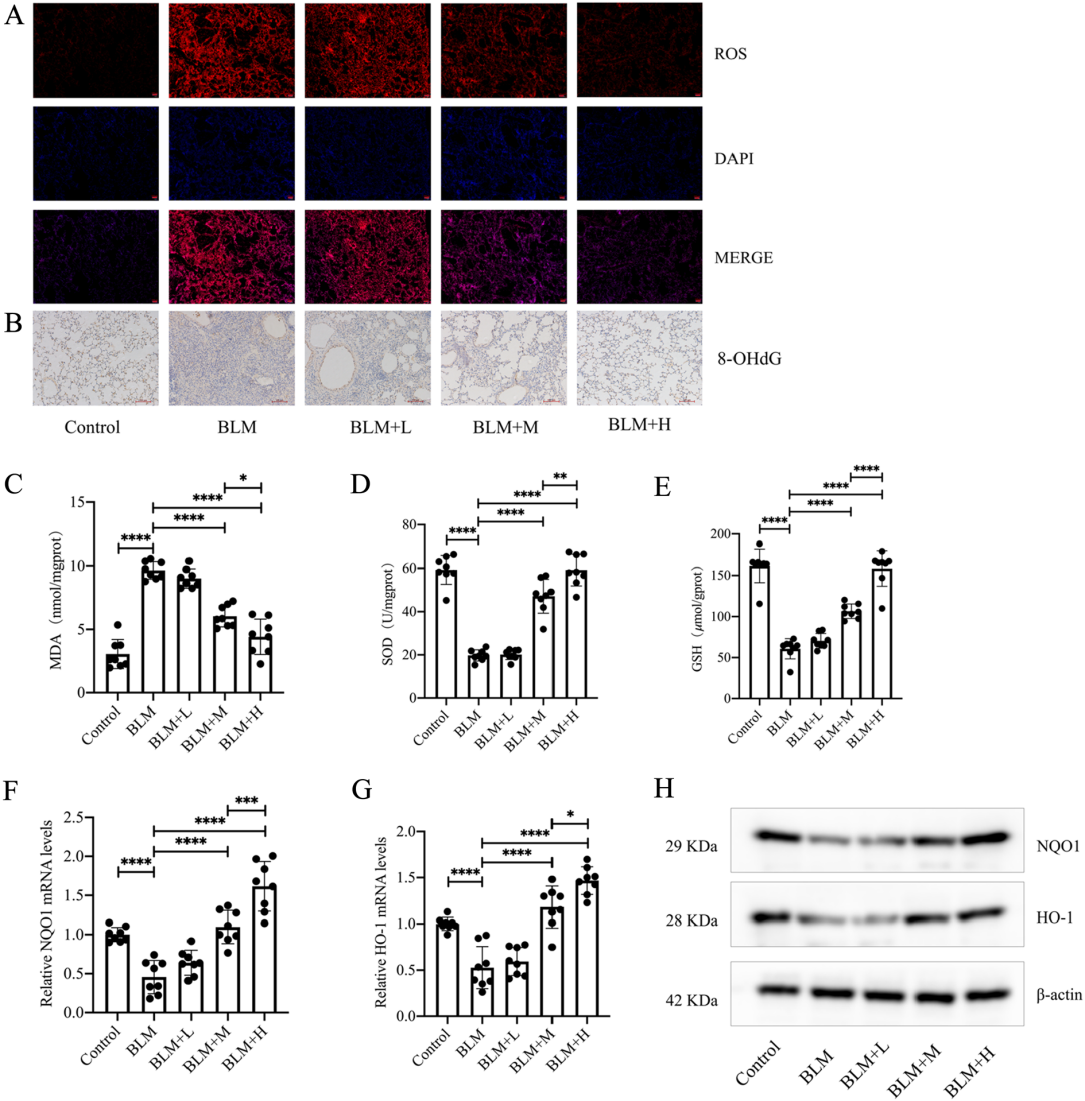
Protodioscin demonstrated a notable reduction in the levels of MDA, 8-OHdG, and ROS, coupled with an elevation in SOD activity and GSH level. Additionally, protodioscin increased the levels of HO-1 and NQO1 in the mouse lung tissues with pulmonary fibrosis. The assessment of ROS level in mouse lung tissues was undertaken through DHE staining (**A**). Immunohistochemical analysis, depicted in images at a magnification of × 100, was utilized to assess the expression of 8-OHdG in mouse lung tissues (**B**). Biochemical assays were utilized to measure MDA and GSH levels, as well as SOD activity (**C**–**E**). It was attempted to determine mRNA and protein levels of HO-1 and NQO1 through qRT-PCR and WB (**F**–**H**). The designations BLM + H, BLM + M, BLM + L, BLM, and Control correspond to the high-dose treatment, medium-dose treatment, low-dose treatment, lung fibrosis model, and control groups, respectively. Expression of data was undertaken in form of mean ± standard deviation. The sample size, particularly per group, was eight, * *P* < 0.05; *It is suggestive of statistical significance at the *P* threshold below 0.05; **Denotes heightened significance at the *P* threshold below 0.01; ***Reflects substantial significance at the *P* threshold below 0.001; ****Demonstrates remarkable significance at the *P* threshold below 0.0001

Protodioscin exerts anti-oxidative stress effects [24], and to verify whether this was linked to its anti-pulmonary fibrosis effects, the ROS contents of lung tissues from the different groups were assessed. This showed that the levels of ROS in fibrotic tissues were markedly reduced at medium and high doses of protodioscin (Fig. 2A). In addition, we examined the levels of 8-OHdG, an oxidative stress marker, in lung tissues using immunohistochemistry and found that these two doses also significantly reduced the levels of 8-OHdG (Fig. 2B). We also examined three other oxidative stress markers in mouse lung tissues using biochemical methods, and the results showed that medium and high doses of protodioscin were effective in increasing GSH levels and SOD activity, as well as decreasing MDA levels (Fig. 2C–E). As the involvement of Nrf2 in counteracting oxidative stress is well-known, we examined whether protodioscin activated Nrf2 in mouse fibrotic lung tissues. In this case, the antioxidant genes *HO-1* and *NQO1*, downstream of Nrf2, were found to be markedly upregulated at moderate and high doses of protodioscin (Fig. 2F–H), indicating that the compound activated Nrf2. These results suggest that protodioscin has an in vivo anti-oxidative stress effect that could be linked to Nrf2 activation.

### Protodioscin inhibits TGF-β1-induced transition of fibroblasts to myofibroblasts

**Fig. 3:**
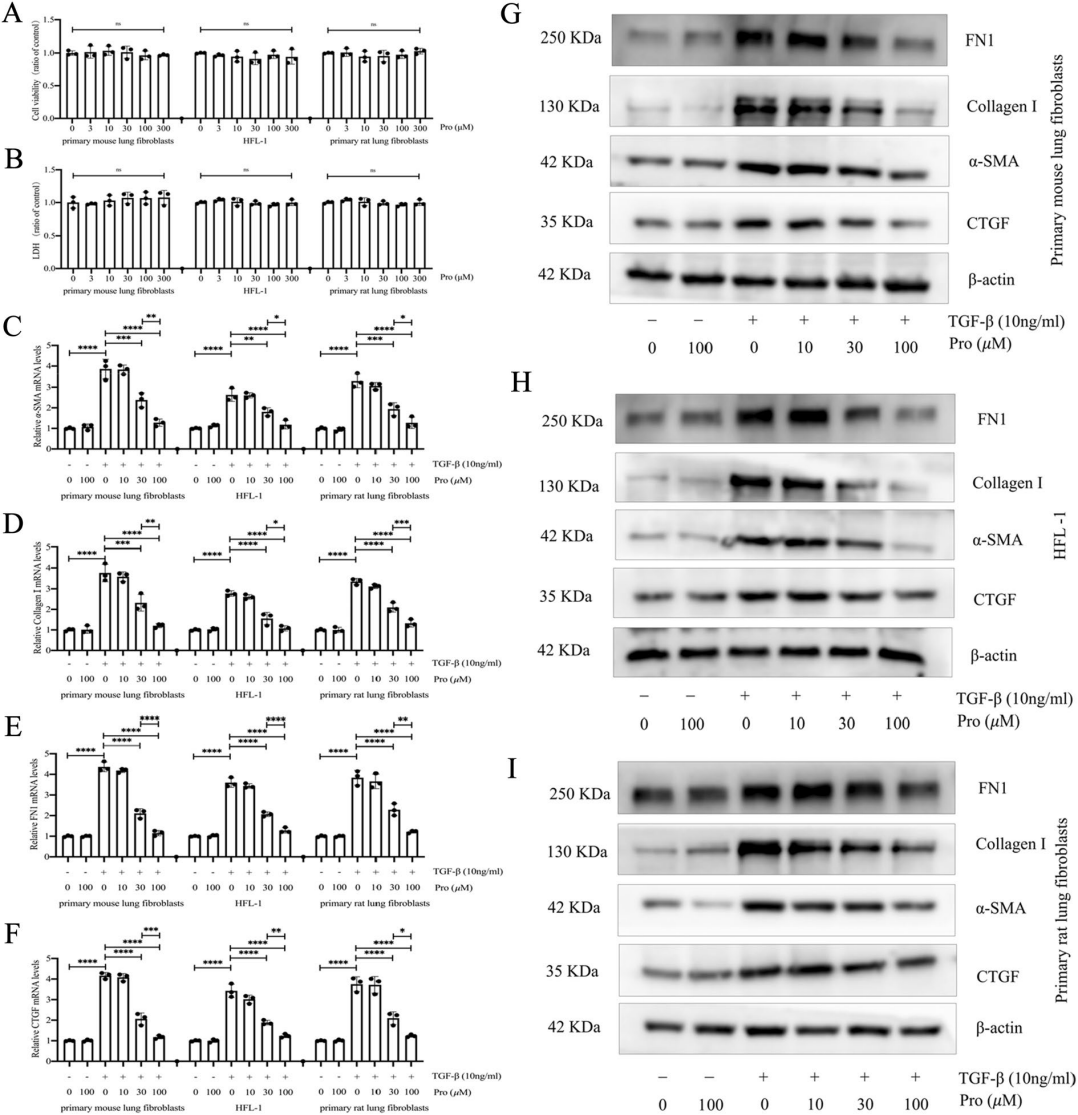
Protodioscin exhibited the capacity to diminish TGF-β1-induced expression levels of α-SMA, FN1, CTGF, and Col-I. The influence of various protodioscin concentrations on the viability of primary rat lung fibroblasts, HFL-1 cells, and primary mouse lung fibroblasts, as well as its effects on LDH levels, were evaluated through biochemical methods (**A**, **B**). It was attempted to co-incubate fibroblasts with diverse concentrations of protodioscin, with or without TGF-β1 (10 ng/mL), for a duration of 24 h. Subsequently, qRT-PCR was employed to quantify the mRNA levels of *α-SMA*, *Col1a1*, *FN1*, and *CTGF* (**C**–**F**). WB was undertaken to analyze the protein levels of α-SMA, Col1a1, FN1 and CTGF (**G**–**I**). Expression of data was undertaken in form of mean ± standard deviation, and the experiments were replicated independently on a minimum of three occasions, * *P* < 0.05; *It is suggestive of statistical significance at the *P* threshold below 0.05; **Denotes heightened significance at the *P* threshold below 0.01; ***Reflects substantial significance at the *P* threshold below 0.001; ****Demonstrates remarkable significance at the *P* threshold below 0.0001

Myofibroblasts are key cells involved in the development of pulmonary fibrosis. To further explore the anti-fibrotic mechanism of protodioscin, we observed its inhibitory effects on TGF-β1-induced transition of primary rat lung fibroblasts, HFL-1 cells and primary mouse lung fibroblasts to myofibroblasts. First, we investigated how varying concentrations of protodioscin (3, 10, 30, 100, and 300 μM) impacted cell viability and LDH levels in the supernatant of the three cell lines. We found that none of the above concentrations of protodioscin had significant effects on cell viability and cell supernatant LDH levels (Fig. 3A, B). By co-incubating various fibroblasts with or without TGF-β1 for 24 h, we found that 30 μM and 100 μM protodioscin significantly decreased TGF-β1-induced protein and mRNA expression of myofibroblast markers col-I, FN1, CTGF, and α-SMA, with the effects of 100 μM being more pronounced than that of 30 μM (Fig. 3C–I). These results indicated that protodioscin effectively inhibited the transition of fibroblasts to myofibroblasts in vitro.

### Protodioscin reduces ROS levels during myofibroblast transition and activates Nrf2

**Fig. 4:**
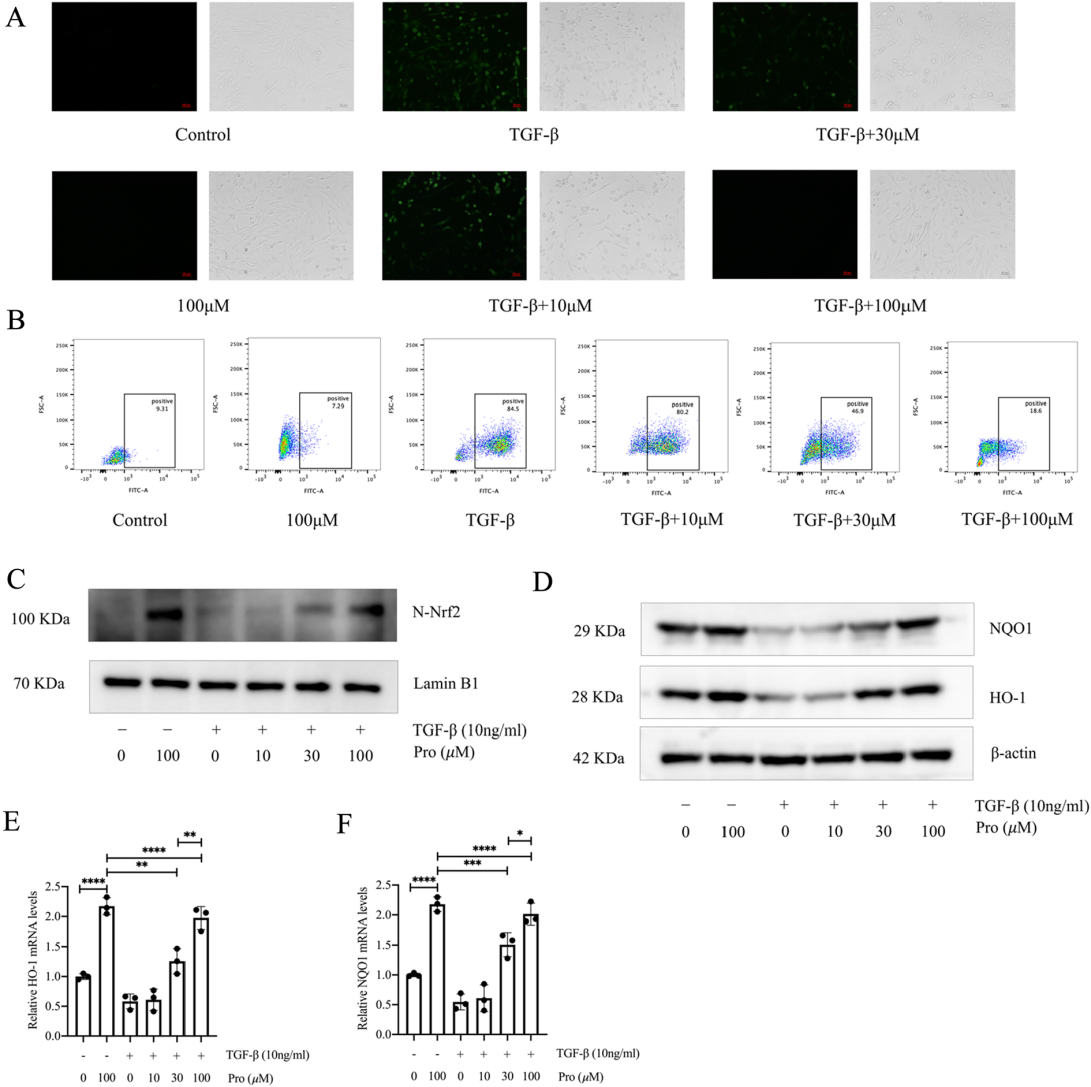
Protodioscin decreased the ROS level and upregulated HO-1 and NQO1 during myofibroblast transition. Primary mouse lung fibroblasts were co-incubated for 24 h with 10 μM, 30 μM, and 100 μM protodioscin with or without TGF-β1 (10 ng/mL). ROS fluorescence probes were used to visualise the level of ROS by microscopy after incubation (**A**). The proportion of ROS-positive cells was detected by flow cytometry (**B**). WB analysis of Nrf2 levels in nuclear proteins in each group (**C**). WB analyzed the protein levels of HO-1 and NQO1 in each group (**D**). Determining the mRNA expression levels of *HO-1* and *NQO1* in each group was undertaken through qRT-PCR (**E**, **F**). Expression of data was undertaken in form of mean ± standard deviation, and the experiments were replicated independently on a minimum of three occasions, * *P* < 0.05; *It is suggestive of statistical significance at the *P* threshold below 0.05; **Denotes heightened significance at the *P* threshold below 0.01; ***Reflects substantial significance at the *P* threshold below 0.001; ****Demonstrates remarkable significance at the *P* threshold below 0.0001

During the development of pulmonary fibrosis, large amounts of ROS are released by myofibroblasts, with this increase in ROS leading to an increase in myofibroblasts, resulting in a feedback loop [27]. In the in vivo experiments, we found that protodioscin reduced ROS and activated Nrf2 in mouse fibrotic lung tissues. To further investigate the mechanism of protodioscin against pulmonary fibrosis, primary lung fibroblasts of mice were co-incubated for 24 h with varying protodioscin concentrations with or without TGF-β1. Immunofluorescence and flow cytometry were then performed to detect the ROS levels in each group. The results showed that 30 μM versus 100 μM protodioscin significantly decreased the ROS level and the proportion of ROS-positive cells (Fig. 4A, B). Meanwhile, we also observed that 30 μM and 100 μM protodioscin significantly increased the nuclear translocation of Nrf2 (Fig. 4C) and significantly upregulated the antioxidant genes *HO-1* and *NQO1* (Fig. 4D–F). These results further suggest that protodioscin may exert its anti-pulmonary fibrosis effect through its anti-oxidative stress effect, and that this anti-oxidative stress effect could be linked to Nrf2 activation.

### Nrf2 mediates protodioscin inhibition of myofibroblast transition

**Fig. 5:**
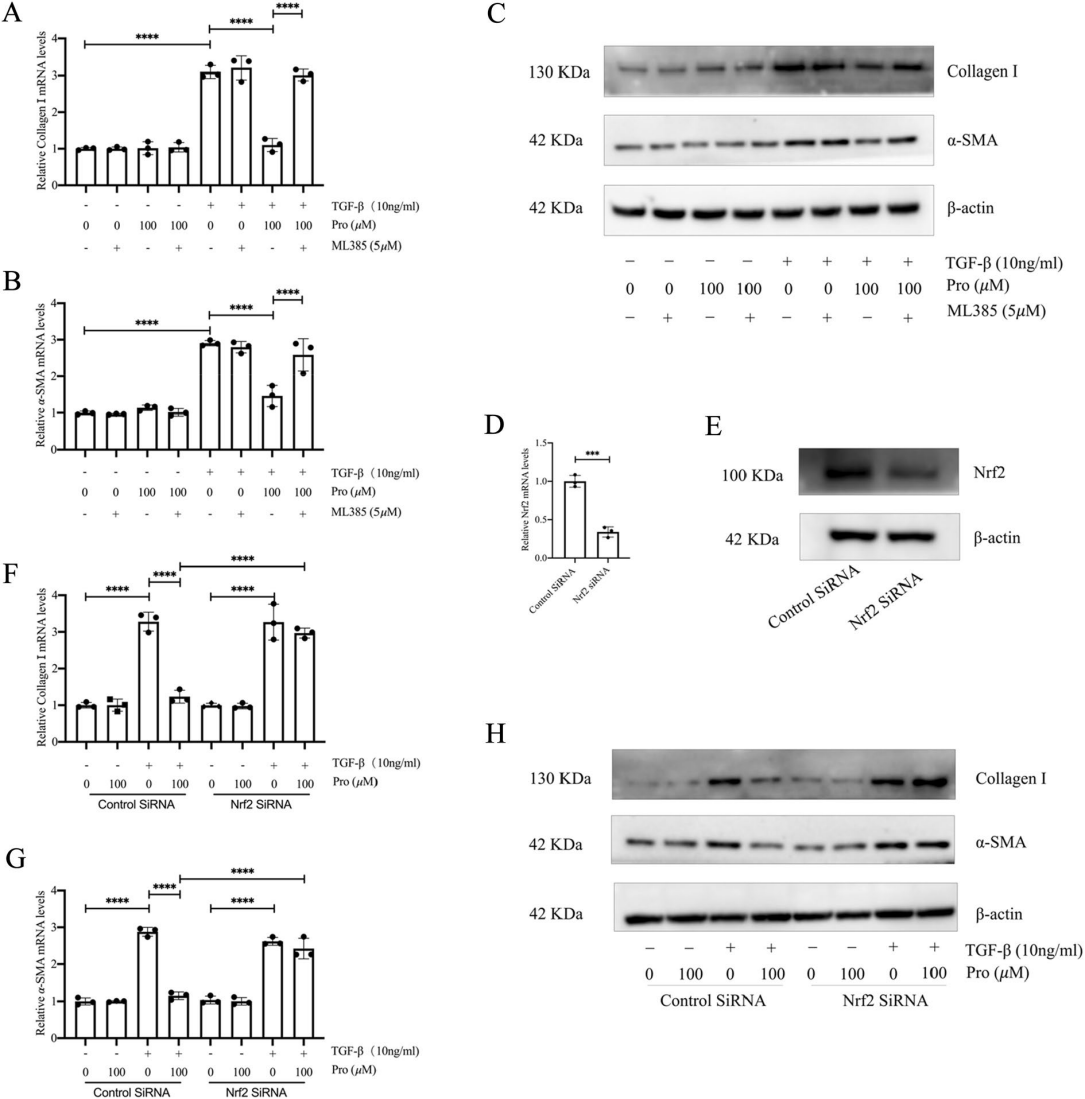
ML385 or Nrf2 silencing inhibited the effects of protodioscin on col-I and α-SMA expression during myofibroblast transition. The level of mRNA expression for *α-SMA* and *Col1a1* in primary mouse lung fibroblasts was determined by qRT-PCR after pretreatment with ML385 for 1 h and co-incubation of protodioscin with TGF-β1 for 24 h (**A**, **B**). WB was undertaken to quantify the expression levels of col-I and α-SMA proteins through WB (**C**). The mRNA and protein levels of Nrf2 were detected by WB and qRT-PCR after Nrf2 silencing (**D**, **E**). The mRNA levels of *α-SMA* and *Col1a1* were detected by qRT-PCR (**F**, **G**). The protein levels of α-SMA and col-I were detected by WB (**H**). Expression of data was undertaken in form of mean ± standard deviation, and the experiments were replicated independently on a minimum of three occasions, * *P* < 0.05; *It is suggestive of statistical significance at the *P* threshold below 0.05; **Denotes heightened significance at the *P* threshold below 0.01; ***Reflects substantial significance at the *P* threshold below 0.001; ****Demonstrates remarkable significance at the *P* threshold below 0.0001

Based on the above results, to further verify whether Nrf2 could mediate the inhibitory effects of protodioscin on myofibroblast transition, we examined the effect of protodioscin on myofibroblast transition using ML385, an inhibitor of Nrf2, or by silencing the *Nrf2* gene before assessing protodioscin’s effects on myofibroblast transition. It was found that a 1-h pretreatment of primary mouse lung fibroblasts with ML385 blocked the effects of protodioscin on the mRNA and protein levels of the TGF-β1-induced myofibroblast markers α-SMA and col-I (Fig. 5A–C). Similarly, after silencing the *Nrf2* gene with SiRNA (Fig. 5D, E), the effects of protodioscin on the mRNA and protein expression levels of col-I and α-SMA were altered (Fig. 5F–H). These results suggest that the prevention of myofibroblast transition by protodioscin was dependent on Nrf2 activation.

### p62 mediates the activation of Nrf2 by protodioscin

**Fig. 6:**
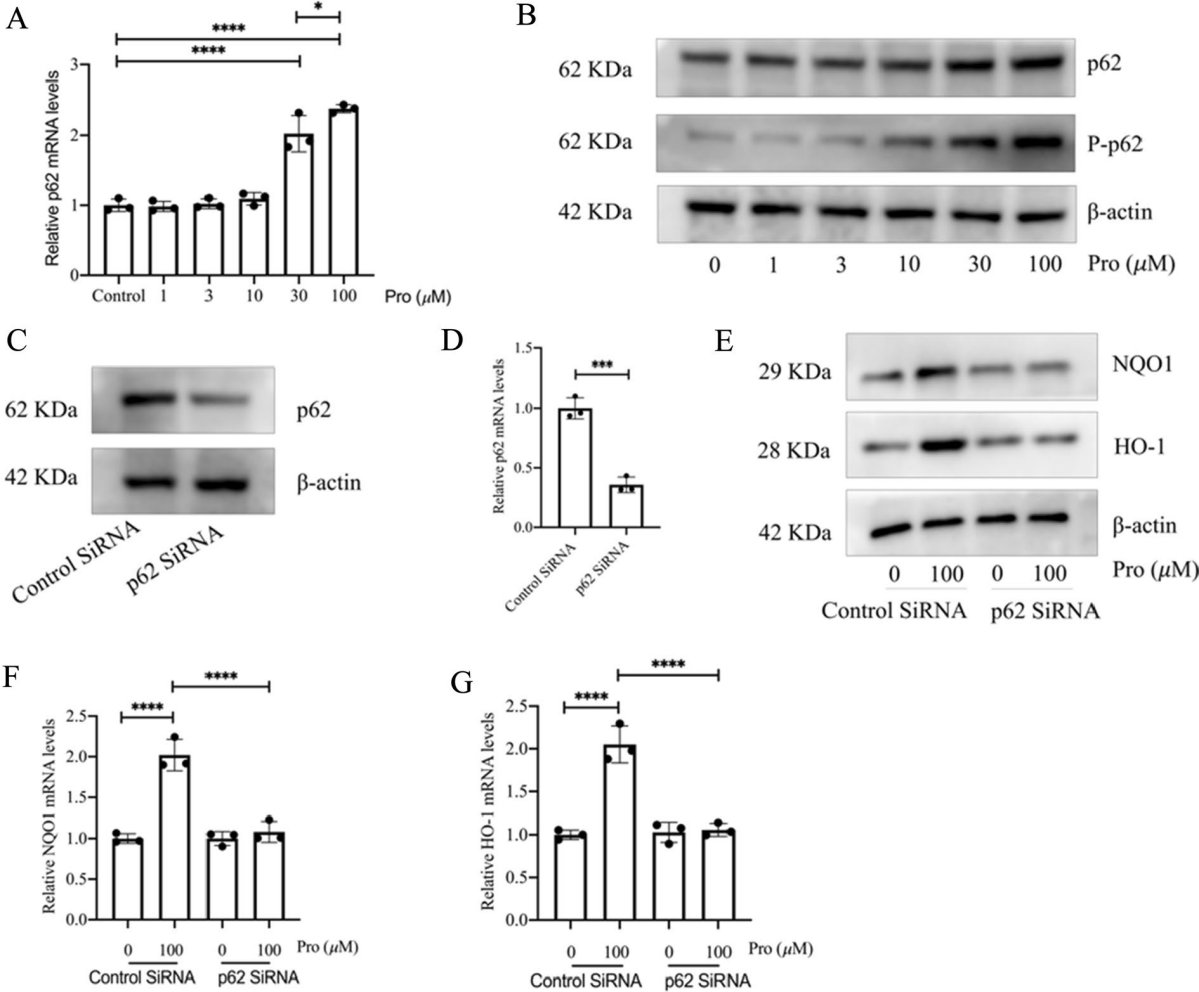
Protodioscin enhanced the expression of p62 and P-p62. The effects of protodioscin on p62 mRNA expression as determined by qRT-PCR (**A**). The effect of protodioscin on the levels of p62 and P-p62 was quantified by WB (**B**). qRT-PCR and WB verified the mRNA and protein levels of p62 after p62 silencing (**C**, **D**). WB and qRT-PCR verified the protein and mRNA levels of HO-1 and NQO1 after p62 silencing (**E**–**G**). Expression of data was undertaken in form of mean ± standard deviation, and the experiments were replicated independently on a minimum of three occasions, * *P* < 0.05; *It is suggestive of statistical significance at the *P* threshold below 0.05; **Denotes heightened significance at the *P* threshold below 0.01; ***Reflects substantial significance at the *P* threshold below 0.001; ****Demonstrates remarkable significance at the *P* threshold below 0.0001

Nrf2 activation is modulated by a variety of proteins. In exploring the activation of Nrf2 by protodioscin, it was observed that protodioscin increased the mRNA and protein expression levels of p62, as well as the phosphorylation of p62 (P-p62) (Fig. 6A, B). After the specific silencing of p62 (Fig. 6C, D), the promotional effect of protodioscin on HO-1 and NQO1’s expression, downstream of Nrf2, was suppressed (Fig. 6E–G), suggesting that p62 could be mediating the activation of Nrf2 through protodioscin. To further investigate the the relationship between p62 and Nrf2 in Nrf2 activation by protodioscin, the Nrf2 gene was knocked down (Additional file 1: Fig. S1A, B), showing that promotion of p62 versus P-p62 was not completely suppressed (Additional file 1: Fig. S1C, D). This result suggests that the effects of protodioscin on p62 expression and phosphorylation depend mainly on other pathways rather than the p62-Nrf2 regulatory loop.

### NBR1 mediates the Nrf2 activation of protodioscin by binding to p62 and Keap1

**Fig. 7:**
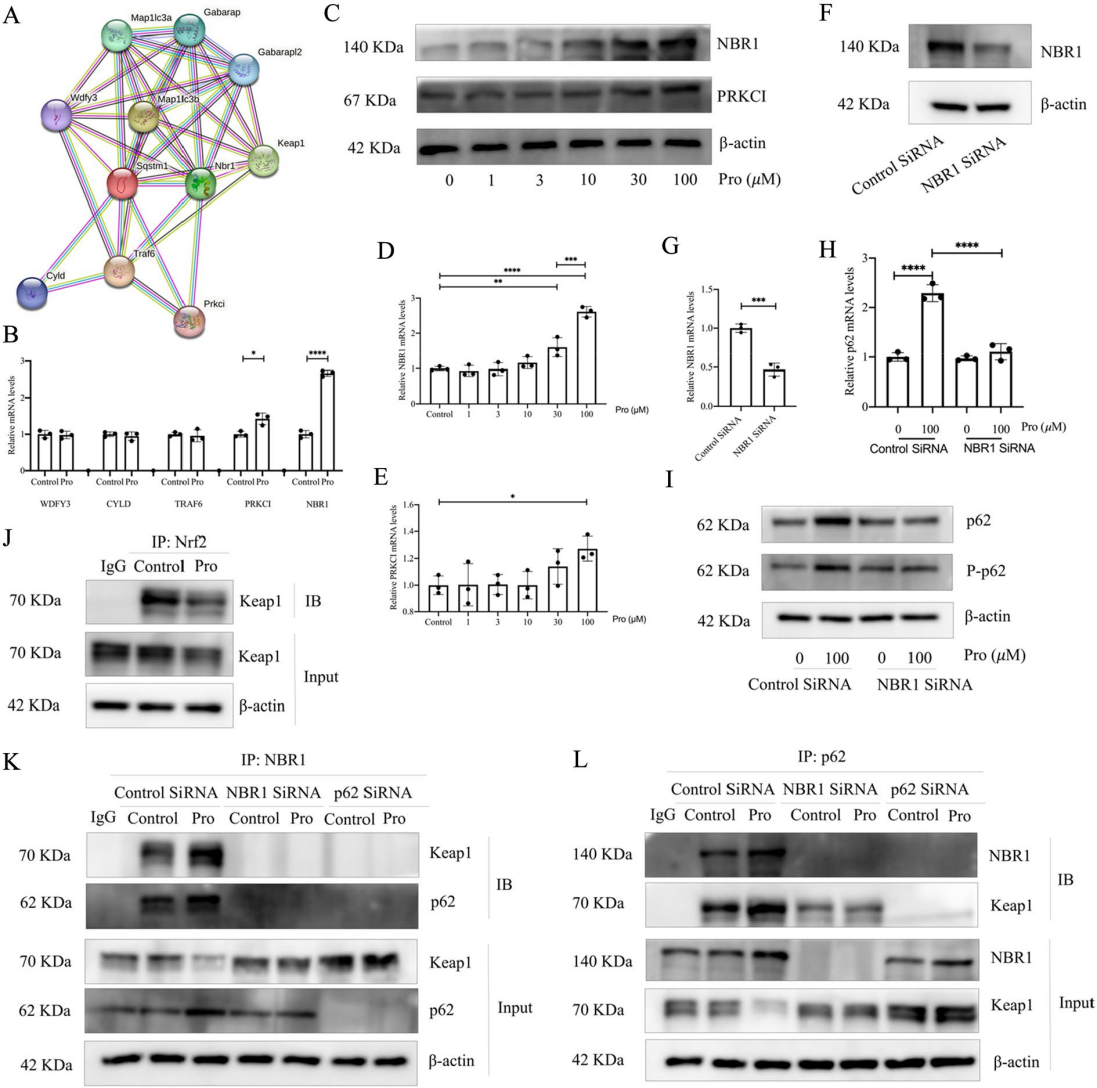
Protodioscin promotes NBR1 expression and binds to p62 and Keap1 to activate Nrf2. p62 direct-acting proteins were predicted using the STRING database (**A**). mRNA expression of *WDFY3*, *CYLD*, *TRAF6*, *NBR1* and *PRKCI* was assessed by qRT-PCR (**B**). The mRNA and protein expression levels of NBR1 and PRKCI after incubation of primary mouse lung fibroblasts with varying concentrations of protodioscin were quantified by WB and qRT-PCR (**C**–**E**). mRNA and protein levels of NBR1 were determined by WB and qRT-PCR after NBR1 silencing (**F**, **G**). The effects of protodioscin on p62 and P-p62 after NBR1 silencing could be indicated by WB and qRT-PCR (**H**, **I**). Co-Ip experiments validate the direct interaction between NBR1, p62 and Keap1 (**J**–**L**). Expression of data was undertaken in form of mean ± standard deviation, and the experiments were replicated independently on a minimum of three occasions, * *P* < 0.05; *It is suggestive of statistical significance at the *P* threshold below 0.05; **Denotes heightened significance at the *P* threshold below 0.01; ***Reflects substantial significance at the *P* threshold below 0.001; ****Demonstrates remarkable significance at the *P* threshold below 0.0001

Based on the above results, to further investigate the effects of protodioscin on p62, we predicted the proteins that have direct interaction with p62 using the STRING database [28] (Fig. 7A). The mRNA expression levels of the genes which may play a role in activating Nrf2 were then examined by qRT-PCR. It was found that protodioscin promoted NBR1 and PRKCI expression (Fig. 7B), and the changes in NBR1 were more pronounced (Fig. 7C–E). Since NBR1 has been shown to promote the activation of Nrf2 [29], to further verify whether NBR1 is a key target of Nrf2 activation by protodioscin by affecting p62, we specifically silenced the NBR1 gene (Fig. 7F, G) and found that the effect of protodioscin on p62 and P-p62 were inhibited (Fig. 7H, I), suggesting that NBR1 is an upstream target of protodioscin affecting p62. To further validate the mechanism by which protodioscin activates Nrf2 by affecting NBR1 and p62, through co-IP experiments, we found that protodioscin led to Nrf2 activation by reducing its binding to Keap1 (Fig. 7J). We further explored the interactions between NBR1, p62 and Keap1 by performing co-IP experiments on cells subjected to different interventions. We found that the activation of Nrf2 was dependent on the action of protodioscin on NBR1 and p62, that is, NBR1, p62, and Keap1 can bind, and protodioscin promotes this effect by affecting p62 through increased expression of NBR1, thus leading to the activation of Nrf2 (Fig. 7K, L), which is a key target for protodioscin to exert its anti-oxidative stress effects. In addition, although the role of p62 in fibrosis has been demonstrated [30, 31], there has been no relevant study exploring the role of NBR1 in fibrosis. Significant associations between the levels of NRB1 and p62 were observed in IPF and control tissues (Additional file 1: Fig. S2), suggesting involvment of NBR1 in lung fibrosis through p62. Thus, NBR1-p62-Nrf2 may be a key target for protodioscin to exert antifibrotic effects by exerting anti-oxidative stress.

### Nrf2 mediates the anti-fibrotic effects of protodioscin in vivo

**Fig. 8:**
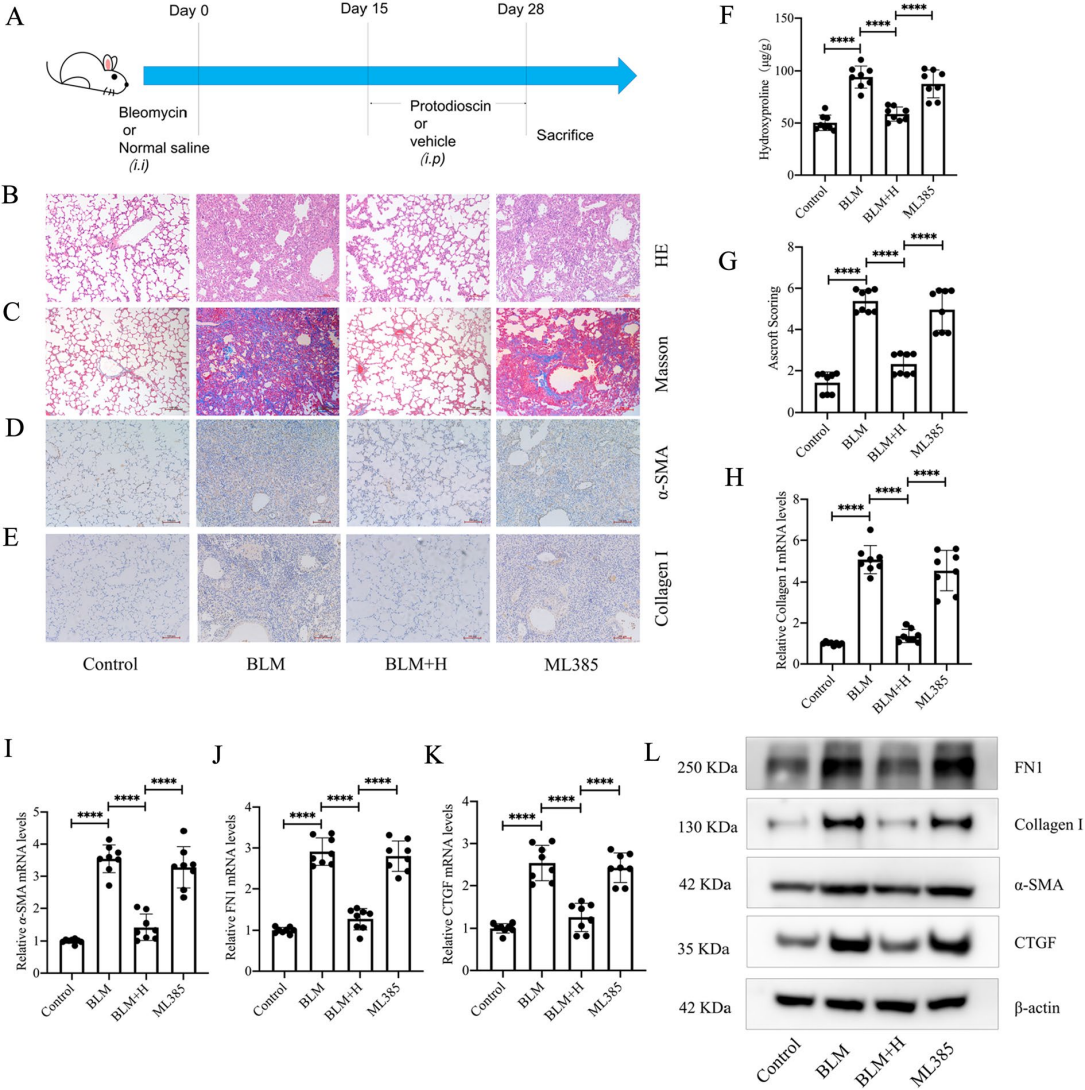
ML385 inhibits the effects of protodioscin on the structure and morphology of lung tissues as well as the levels of lung fibrosis markers in mice with pulmonary fibrosis. A mouse lung fibrosis model was generated through tracheal injections of bleomycin, and 30 mg/kg of ML385 was injected intraperitoneally 3 h prior to the administration of high-dose protodioscin. Its effect on the therapeutic effect of protodioscin was then observed (**A**). Changes in lung tissue structures and extracellular matrix were visualized by HE and Masson staining (magnification × 100) (**B**, **C**) before analyzing the degree of pulmonary fibrosis in each group of mice with the Ashcroft score (**G**). The hydroxyproline contents of lung tissues were determined by biochemical methods (**F**). The positive expression areas of col-I and α-SMA in lung tissues were analyzed by immunohistochemical methods (magnification × 100) (**D**, **E**). Determining the mRNA expression levels of *col1a1*, *FN1*, *CTGF* and *α-SMA* in lung tissues was undertaken through qRT-PCR (**H**–**K**), while the protein levels of col-I, FN1, CTGF and α-SMA were quantified by WB (**L**). ML385 indicates the group treated with ML385 prior to high-dose treatment; BLM + H indicates the high-dose treatment group; BLM indicates the lung fibrosis model group; Control indicates the control group. Expression of data was undertaken in form of mean ± standard deviation. The sample size, particularly per group, was eight, * *P* < 0.05; *It is suggestive of statistical significance at the *P* threshold below 0.05; **Denotes heightened significance at the *P* threshold below 0.01; ***Reflects substantial significance at the *P* threshold below 0.001; ****Demonstrates remarkable significance at the *P* threshold below 0.0001

Based on the outcomes of the in vitro assays described above, we further verified whether protodioscin ameliorated pulmonary fibrosis through the activation of Nrf2 following the in vivo injection of ML385. The administration scheme was to inject ML385 (30 mg/kg) intraperitoneally 3 h prior to the high-dose protodioscin treatment, and then to compare the differences in various fibrosis indices between this group of mice and the high-dose treatment group of mice (Fig. 8A). Results for the morphology of lung tissues and deposition of extracellular matrix, as assessed by HE and Masson staining, showed that ML385 inhibited the effects of protodioscin (Fig. 8B, C). The results of the Ashcroft score and hydroxyproline levels were similarly affected by ML385 (Fig. 8F, G). Immunohistochemical results showed that ML385 also inhibited protodioscin’s ability to reduce the positive expression regions of col-I and α-SMA in the lung tissues of fibrotic mice (Fig. 8D, E). Similarly, ML385 inhibited protodioscin’s effects on the mRNA and protein expression of col-I, FN1, CTGF and α-SMA (Fig. 8H–L). This suggests that Nrf2 mediates the in vivo antifibrotic effects of protodioscin.

### Nrf2 mediates anti-oxidative stress actions of protodioscin in vivo

**Fig. 9:**
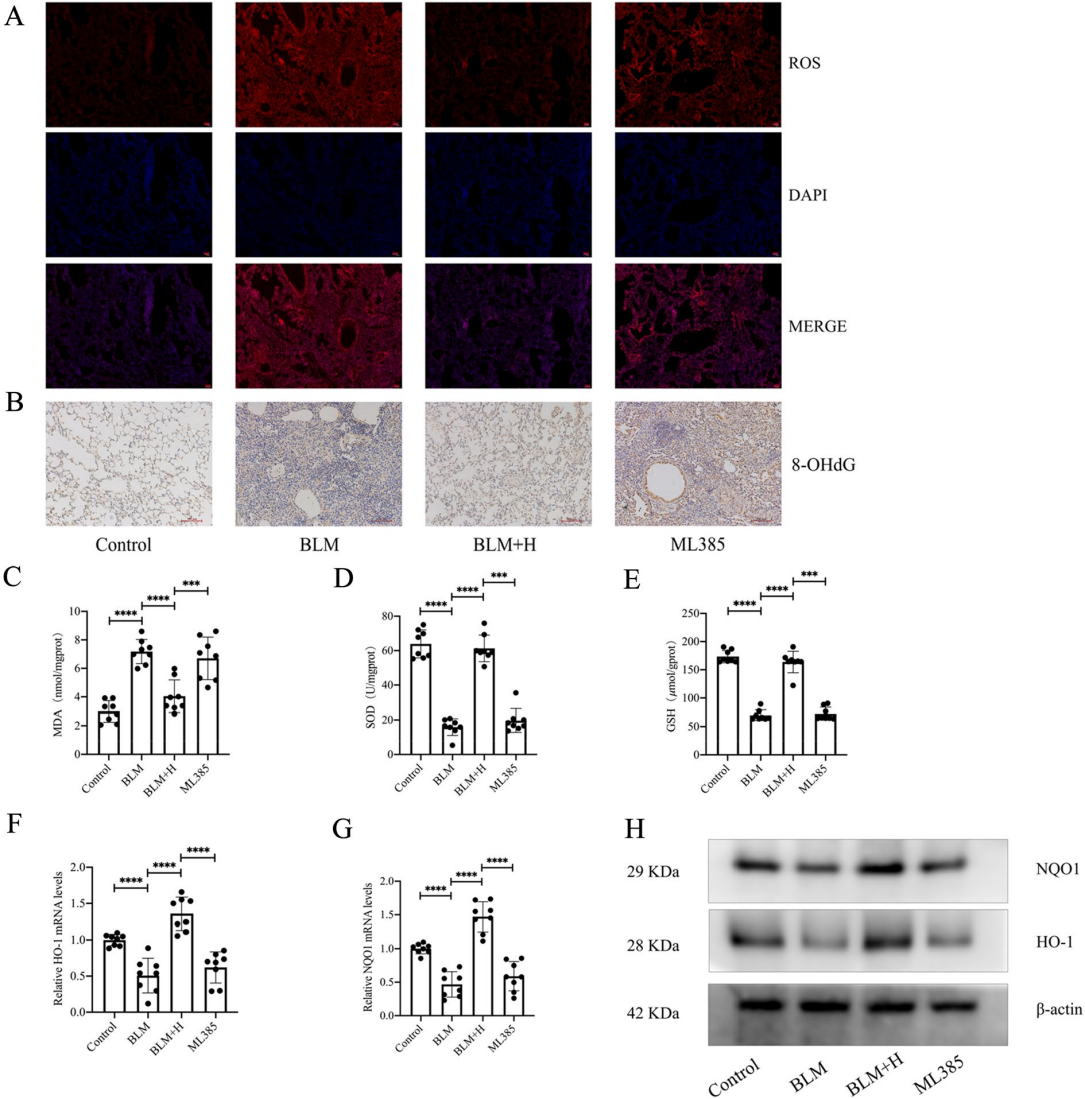
In the context of evaluating the therapeutic influence of protodioscin on pulmonary mouse fibrosis, discernible alterations were noted in the levels of ROS and various oxidative stress markers, involving 8-OHdG, MDA, SOD, and GSH, within the pulmonary tissues of fibrotic mice. The levels of ROS in lung tissues of mice in each group were detected by DHE staining (**A**). Immunohistochemical methods were used to detect the expression level of 8-OHdG in mouse lung tissues (magnification × 100) (**B**). MDA and GSH levels as well as SOD activity were biochemically assessed (**C**–**E**). qRT-PCR and WB were used to assess the level of mRNA and protein expression for HO-1 and NQO1 (**F**–**H**). ML385 indicates the group treated with ML385 prior to high-dose treatment; BLM + H indicates the high-dose treatment group; BLM indicates the lung fibrosis model group; Control indicates the control group. Expression of data was undertaken in form of mean ± standard deviation. The sample size, particularly per group, was eight, * *P* < 0.05; *It is suggestive of statistical significance at the *P* threshold below 0.05; **Denotes heightened significance at the *P* threshold below 0.01; ***Reflects substantial significance at the *P* threshold below 0.001; ****Demonstrates remarkable significance at the *P* threshold below 0.0001

While it was found that ML385 inhibited the in vivo anti-pulmonary fibrosis effect of protodioscin, we also observed an effect of ML385 on the anti-oxidative stress effect of protodioscin. The results showed that ML385 also inhibited protodioscin’s effects in reducing ROS levels in mouse lung tissues (Fig. 9A). Moreover, the effects of protodioscin on several oxidative stress markers, including 8-OHdG, MDA, SOD, and GSH, were inhibited by ML385 (Fig. 9B–E). The promoting effect of protodioscin on the expression of the antioxidant genes HO-1 and NQO1, that are downstream of Nrf2, were similarly inhibited by ML385 (Fig. 9F–H). This suggests that Nrf2 mediates the in vivo anti-oxidative stress effects of protodioscin.

## Discussion

Our study demonstrated, for the first time, that protodioscin exerts an anti-oxidative stress effect by promoting NBR1 expression. This promotion increases the expression and phosphorylation of p62, subsequently activating Nrf2. This anti-oxidative stress activity enables protodioscin to exert anti-fibrotic pharmacological effects.

Oxidative stress plays a key role in IPF’s development [32] as various cytokines, including TGF-β produced in response to endogenous or exogenous oxidative stress stimuli, can induce myofibroblasts formation, leading to the development of IPF [8]. In contrast, myofibroblasts are also the main source of ROS in the process of pulmonary fibrosis, and the large amounts of ROS produced can induce the expression and secretion of various pro-fibrotic factors, including TGF-β, in a number of cells, which in turn forms a vicious circle and facilitates developing pulmonary fibrosis [27]. In the course of figuring out the therapeutic influence of protodioscin on pulmonary fibrosis, substantial variations were discerned in the levels of ROS and various oxidative stress markers, encompassing 8-OHdG, MDA, SOD, and GSH, within the pulmonary mouse tissues accompanied by pulmonary fibrosis. These observations underscore a robust interconnection between protodioscin's anti-pulmonary fibrosis properties and its capacity to ameliorate oxidative stress. Notably, the mitigation of ROS levels has been demonstrated to impede the TGF-β-induced transition of fibroblasts into myofibroblasts [33, 34]. Our in vivo assays also revealed that protodioscin can reduce the levels of ROS during the myofibroblast transition process. This further demonstrates that the anti-oxidative stress effect of protodioscin is an important molecular mechanism for the anti-fibroblastic effect of protodioscin. Furthermore, this mechanism may underlie other pharmacological effects of protodioscin, such as its anti-bladder cancer and diabetic nephropathy improvement effects.

In response to the adverse effects of oxidative stress, the body activates its antioxidant system which is largely regulated by Nrf2 [9]. Various Nrf2 activators have been shown to exert anti-pulmonary fibrosis effects by activating Nrf2 [35–38]. This work showed, for the first time, that protodioscin activates Nrf2 in primary mouse lung fibroblasts, resulting in the increased expression of downstream antioxidant genes. In contrast, the effects of protodioscin on the transition of fibroblasts to myofibroblasts were suppressed after silencing the *Nrf2* gene. This was particularly noted when intraperitoneal injections of the Nrf2 inhibitor ML385 prior to the administration of protodioscin suppressed the latter’s therapeutic effects in mice with pulmonary fibrosis. In addition, we found that Nrf2 mediates the inhibitory effect of protodioscin on the TGF-β/Smad pathway (Additional file 2: Fig. S3). Thus, Nrf2 mediates the anti-oxidative stress and anti-pulmonary fibrosis effects of protodioscin.

p62 is involved in inhibiting fibrotic diseases’ progression, but its exact mechanism is currently unknown [39]. Our previous study showed that the cyclic relationship between p62 and Nrf2 may be a key target for inhibiting pulmonary fibrosis [40]. In this work, we observed, for the first time in primary mouse lung fibroblasts, an elevating effect of protodioscin on both the expression and phosphorylation levels of p62. Moreover, the activation of Nrf2 by protodioscin was inhibited when the p62 gene was silenced, which suggests that the activation of Nrf2 by protodioscin is dependent on p62. Interestingly, even upon silencing the *Nrf2* gene, the elevation effect of protodioscin on the p62 expression and phosphorylation persisted. This implies that protodioscin's effect on p62 goes beyond merely promoting the p62-Nrf2 loop. Using the STRING database, we found that among the proteins that may have a direct correlative effect on Nrf2 activation by p62, the expression of NBR1 and PRKCI increased upon the addition of protodioscin, with protodioscin having a more pronounced effect on NBR1 than on PRKCI. NBR1, a receptor for selective autophagy that acts synergistically with p62, has also been shown to activate Nrf2 by affecting p62; however, the detailed mechanism is currently unknown [29, 41]. In our study, the expression and phosphorylation of p62 increased by protodioscin were inhibited by specific silencing of the NBR1 gene, suggesting that activation of the p62-Nrf2 loop by protodioscin is dependent on NBR1. Through co-IP experiments, we found for the first time that, in primary mouse lung fibroblasts, NBR1 not only promotes p62 expression and its subsequent phosphorylation but also promotes the binding of p62 to Keap1 by binding to p62, which leads to a decrease in Nrf2 binding to Keap1, thus increasing Nrf2 activation. This suggests that NBR1-p62-Nrf2 is a key target for the anti-oxidative stress effect of protodioscin and its anti-pulmonary fibrosis effect and may also serve as novel but promising drug target for treating pulmonary fibrosis. In addition, our study also revealed that protodioscin promotes the expression of another protein directly related to p62, PRKCI, which is also a receptor for selective autophagy, and p62, which also plays a synergistic role in autophagy [42]. PRKCI has also been shown to correlate with the activation of Nrf2, although the detailed mechanism is unknown [43]. Similar to NBR1, PRKCI can directly bind to the PB1 structural domain of p62 through its PB1 structural domain and affect its LIR structural domain of p62 [44]. Therefore, we speculated that PRKCI may also activate Nrf2 by affecting its binding to Keap1 through binding to p62, similar to NBR1. This proposition awaits further verification in future studies.

Protodioscin has also been reported to exert its pharmacological effects through its effect on the MPAK pathway; however, its detailed mechanism remains unclear [22, 45]. Crosstalk exists between the MAPK and Nrf2 pathways, with the MAPK pathway being crucial for the development of pulmonary fibrosis [46–48]. Hence, investigating whether the Nrf2-mediated antifibrotic effect of protodioscin is linked to the inhibition of the MAPK pathway as well as the activation of the antioxidant system, warrants further exploration. According to our study, protodioscin inhibits oxidative stress through the NBR1-p62-Nrf2 pathway and, as such, it exerts an anti-pulmonary fibrosis effect. We therefore propose this pathway as a novel drug target for treating pulmonary fibrosis. This proposition holds significant theoretical value by shedding light on a novel molecular mechanism underlying pulmonary fibrosis treatment. Moreover, from a clinical perspective, targeting the NBR1-p62-Nrf2 pathway could potentially lead to the development of innovative therapeutic strategies for managing pulmonary fibrosis, addressing a critical medical need.

## Conclusion

The mechanism through which protodioscin exerts its anti-oxidative stress and anti-fibrosis effects was determined by studying the link between NBR1, Nrf2 and p62. Protodioscin promoted the expression and phosphorylation of p62 by increasing the expression of NBR1, which led to an increase in the binding of NBR1 to p62 and Keap1, resulting in increased nuclear translocation of Nrf2. This increased translocation upregulated the antioxidant genes HO-1 and NQO1, thus inhibiting oxidative stress to exert antifibrotic effects. Our results suggest that protodioscin is of therapeutical potential for treating pulmonary fibrosis, and, in this context, we suggest that the NBR1-p62-Nrf2 pathway represents a promising therapeutic target that can be further explored.

### Supplementary Information

**Additional file 1: Fig. S1.** Effect of silencing Nrf2 on the expression and phosphorylation levels of protodioscin on p62. **Fig. S2.** Spearman's correlation analysis of p62 gene and NBR1 gene in the lungs of IPF patients and controls .

**Additional file 2: Fig. S3.** Nrf2 mediated the inhibitory effect of protodioscin on the TGF-β/Smad pathway.

## Data Availability

The datasets utilized in this investigation, as well as the analytical findings, are accessible through formal solicitation from the corresponding author. A comprehensive compilation of all data generated or scrutinized during the research is provided within this article and its accompanying Additional information files.

## Abbreviations

IPF: Idiopathic pulmonary fibrosis
Nrf2: Nuclear factor erythroid 2-related factor 2
Keap1: Kelch-like ECH-associated protein 1
HO-1: Heme oxygenase 1
NQO1: NAD(P)H dehydrogenase, quinone 1
ROS: Reactive oxygen species
MDA: Malondialdehyde
SOD: Superoxide dismutase
GSH: Glutathione
LDH: Lactate dehydrogenase
CKK-8: Cell counting kit-8
qRT-PCR: Quantitative real-time polymerase chain reaction
WB: Western blotting
co-IP: Co-immunoprecipitation
HE: Hematoxylin and eosin
8-OHdG: 8-Hydroxy-2-deoxyguanosine
HFL-1: Human fetal lung fibroblast 1 cells
P-p62: Phosphorylation of p62
FN1: Fibronectin 1
