# Supplementary material for: NBR1-p62-Nrf2 mediates the anti-pulmonary fibrosis effects of protodioscin
Source: Chin Med. 2024 Apr 8;19:60. doi: 10.1186/s13020-024-00930-0 (PMC11003024; doi:10.1186/s13020-024-00930-0)
Supplement: Supplementary file 1 — Additional file 1: Fig. S1. Effect of silencing Nrf2 on the expression and phosphorylation levels of protodioscin on p62. Fig. S2. Spearman's correlation analysis of p62 gene and NBR1 gene in the lungs of IPF patients and controls . [file 13020_2024_930_MOESM1_ESM.pdf]

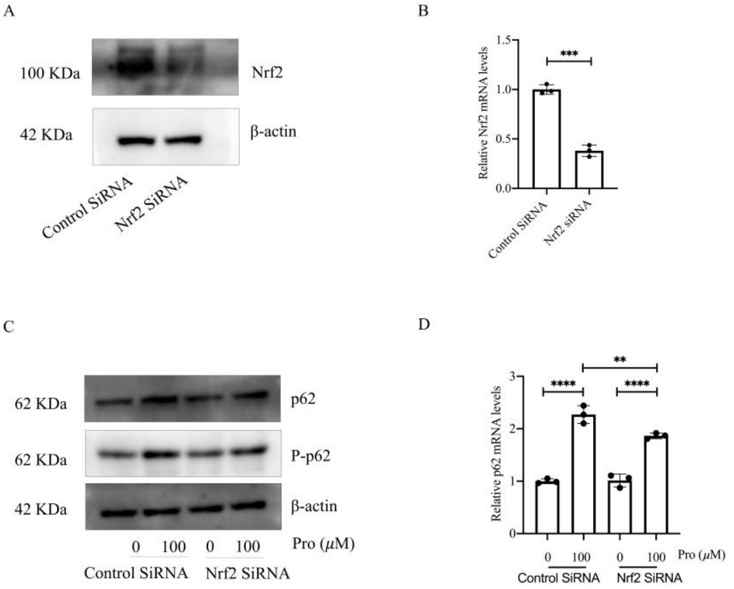

Supplementary Fig. 1 Effect of silencing Nrf2 on the expression and phosphorylation levels of protodioscin on p62. Quantitative Real-Time Polymerase Chain Reaction (Q-PCR) and Western blotting (WB) verified the mRNA and protein expression levels of Nrf2 after Nrf2 silencing (A, B). WB was performed to determine the effect of Nrf2 silencing of protodioscin on the protein expression of p62 and P-p62 (C). Q-PCR was performed to detect the effect of Nrf2 silencing of protodioscin on the mRNA expression of p62 (D). Data are expressed as mean ± standard deviation, and all experiments were repeated independently at least three times, *$P < 0.05$; ** $P < 0.01$; *** $P < 0.001$; **** $P < 0.0001$.

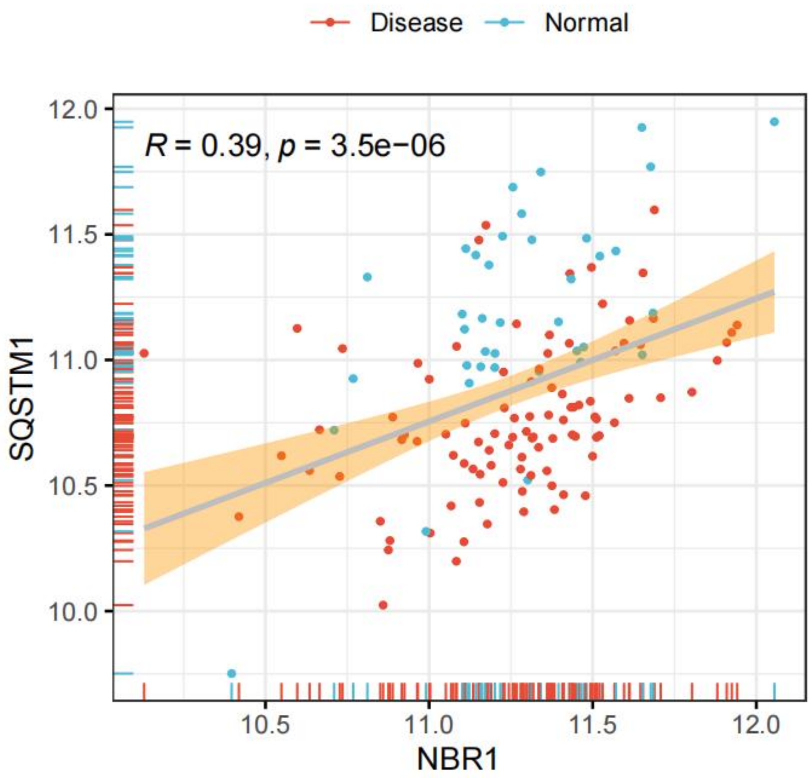

Supplementary Fig. 2 Spearman's correlation analysis of p62 gene and NBR1 gene in the lungs of IPF patients and controls, which were significantly correlated.
