# Supplementary material for: NBR1-p62-Nrf2 mediates the anti-pulmonary fibrosis effects of protodioscin
Source: Chin Med. 2024 Apr 8;19:60. doi: 10.1186/s13020-024-00930-0 (PMC11003024; doi:10.1186/s13020-024-00930-0)
Supplement: Supplementary file 2 — Additional file 2: Fig. S3. Nrf2 mediated the inhibitory effect of protodioscin on the TGF-β/Smad pathway. [file 13020_2024_930_MOESM2_ESM.pdf]

A

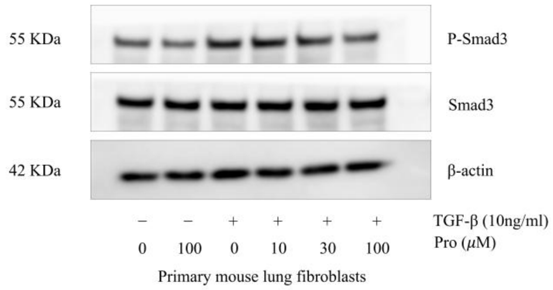

B

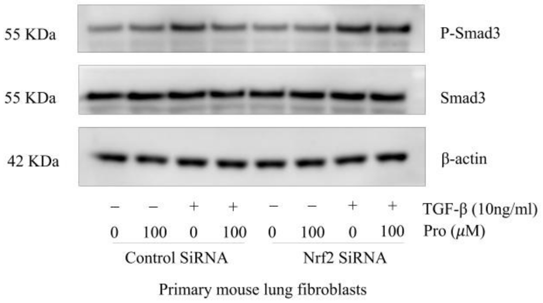

Supplementary Fig. 3 Nrf2 mediated the inhibitory effect of protodioscin on the TGF-β/Smad pathway. Western blotting (WB) detection of the effect of different concentrations of protodioscin on the phosphorylation level of Smad3 (A). WB detection of the effect of protodioscin on the phosphorylation level of Smad3 after Nrf2 silencing (B).
